# A new species record of *Hemiphyllodactylus lungcuensis* Luu, Nguyen, Do, Pham, Hoang, Nguyen, Le, Ziegler, Grismer & Grismer, 2023 (Squamata, Gekkonidae) from Yunnan Province in China

**DOI:** 10.3897/BDJ.13.e173469

**Published:** 2025-12-17

**Authors:** Zhang Dongru, Zhang Leilei, Liu Shunfeng, Liu Guangbin, Fan Yaling, Shen Wenjing, Chen Zhengpeng, Han Keguo, Li Jianhong, Zhou Hongxin

**Affiliations:** 1 Liupanshui Normal University, Liupanshui, China Liupanshui Normal University Liupanshui China; 2 Yunnan Open University, Kunming, China Yunnan Open University Kunming China; 3 Bureau of Wenshan National Nature Reserve, Wenshan, China Bureau of Wenshan National Nature Reserve Wenshan China; 4 Kunming Institute of Zoology, Chinese Academy of Sciences, Kunming, China Kunming Institute of Zoology, Chinese Academy of Sciences Kunming China; 5 Hainan University, Sanya, China Hainan University Sanya China

**Keywords:** Funing County, morphology, ND2, new record, slender gecko

## Abstract

**Background:**

*Hemiphyllodactylus lungcuensis* Luu, Nguyen, Do, Pham, Hoang, Nguyen, Le, Ziegler, Grismer & Grismer was described in 2023, based on seven specimens. Its type locality is Lung Cu Commune, Dong Van District, Ha Giang Province, in north-eastern Vietnam, near the Sino-Vietnamese border. Currently, the species is known only from the type locality; there are no records of its occurrence in China.

**New information:**

On 10 August 2025, we collected a single *Hemiphyllodactylus* specimen in Lida Town, Funing County, Yunnan Province, China. In the phylogenetic tree, this individual clusters with the paratypes and holotype of *H. lungcuensis* with strong support. The specimen from China agrees well with the original morphological description of the species and has a minor mean genetic distance of 1.9% in the ND2 gene with the type specimen of this species. Here, we report the first record of *H. lungcuensis* for China.

## Introduction

*Hemiphyllodactylus* is one of the fastest-growing genera in the family Gekkonidae in recent years, comprising 72 species (Uetz et al. 2025) and is mainly distributed in South Asia, Southeast Asia and the Indo–Pacific islands (Zug 2010, Grismer et al. 2013, Grismer et al. 2018, Agarwal et al. 2019, Eliades et al. 2019, Agung et al. 2021, Agung 2022). Grismer et al. (2013) incorporated molecular data into their study of *Hemiphyllodactylus* and classified the species into two species groups: the *harterti* group and *typus* group, the latter group, in turn, encompassing seven clades.

In China, 23 species of *Hemiphyllodactylus* have been recorded. According to recent genus–wide molecular phylogenetic studies, all *Hemiphyllodactylus* species in China belong to the *typus* group and they are divided into five clades (Grismer et al. 2013, Grismer et al. 2014a, Grismer et al. 2014b, Tri et al. 2014, Grismer et al. 2017, Grismer et al. 2018, Grismer et al. 2020, Grismer 2020, Agung et al. 2021, Zhou et al. 2024a, Zhou et al. 2024b, Zhou et al. 2025a, Zhou et al. 2025b, Wang et al. 2025): clade 1 (*H. typus*) is a widely distributed species and distributed in Hainan Island and Taiwan Island in China; both clade 3 (*H. longlingensis*, *H. zhutangxiangensis*, *H. zayuensis*, *H. changningensis*, *H. gengmaensis*, *H. laowozhenensis* and *H. jinghongensis*) and clade 4 (*H. jinpingensis*, *H. simaoensis*, *H. mengsongcunensis*, *H. menglianensis*, *H. diaoluoshanensis* and *H. jianfenglingensis*) of Agung (2022) colonised China from western Indochina and both clade 6 (*H. dushanensis*, *H. hongkongensis*, *H. dupanglingensis*, *H. zugi*, *H. huishuiensis*, *H. yanshanensis*, *H. guangnanensis* and *H. lingshuiensis*) and clade 7 (*H. yunnanensis*) of Agung (2022) colonised China from eastern Indochina.

Recently, during our reptile survey in Yunnan Province, we discovered a specimen belonging to *Hemiphyllodactylus*. Based on morphological and genetic evidence, it has been determined that this specimen should be assigned to *H. lungcuensis*. This represents the first record of the species in China.

## Materials and methods

**Sampling**

**Figure 1.**
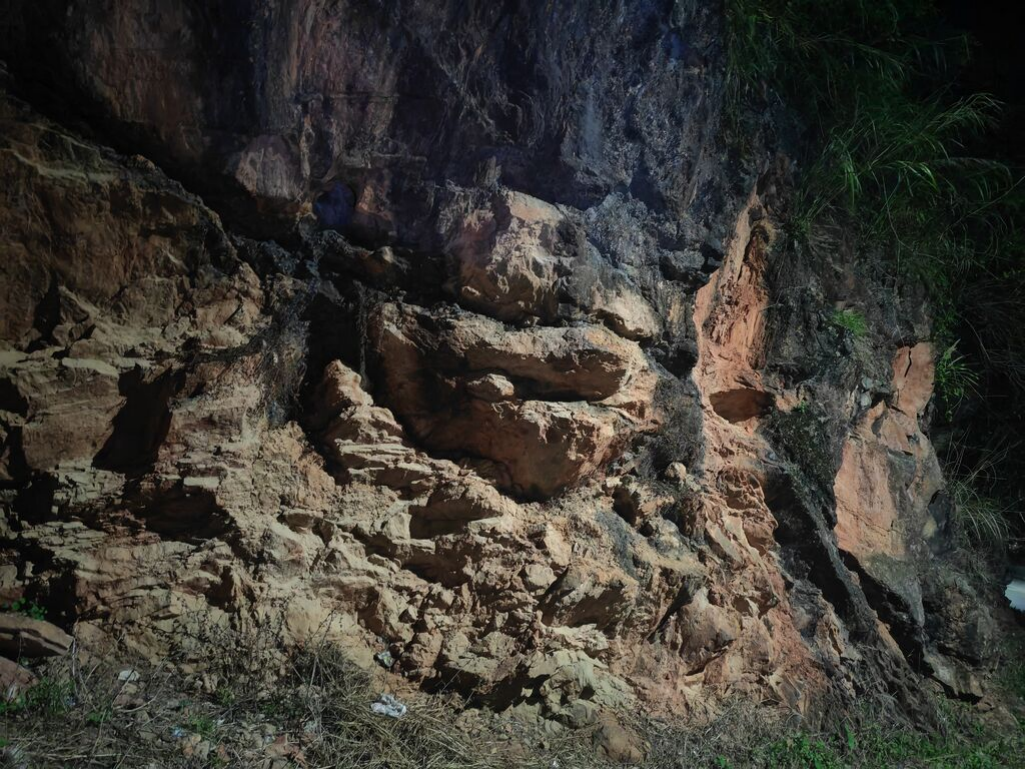
Habitat of *Hemiphyllodactylus lungcuensis* in Lida Town, Funing County, Yunnan, China.

A specimen (LPSZDR0141) was collected from Lida Town, Funing County of Yunnan Province, China on 10 August 2025 (Fig. 1). The specimen was preserved in 80% ethanol and its liver tissue was preserved in 95% ethanol. The specimen was deposited in Liupanshui Normal University (LPSSY).

**Molecular data and phylogenetic analyses**

We used Trelief Hi–Pure Animal Genomic DNA Kit for genomic DNA extraction following the manufacturer’s protocol (https://www.tsingke.com.cn). We amplified and sequenced the complete mitochondrial NADH dehydrogenase subunit 2 gene (ND2), totalling 1,038 bp using the primers L4437b and H5934 (Macey et al. 1997). The protocol for polymerase chain reaction (PCR) amplifications followed Agung et al. (2021). Genomic DNA extraction, PCR processes and sequencing were executed at Beijing Tsingke Biotechnology Co., Ltd. The specimen sequences have been deposited in GenBank, with accession number PX398552.

A total of 72 ND2 sequences were retrieved from GenBank, including two outgroup sequences (*Hemiphyllodactylus harterti*) and 70 from extant *Hemiphyllodactylus* species; together with our newly-generated sequence, they are listed in Table 1. Sequences were assembled and manually proofread in SeqMan (DNASTAR, Inc., Madison, WI, USA), then aligned using Clustal W (Thompson et al. 1994), implemented in MEGA 7 (Kumar et al. 2016). For phylogenetic relationship analysis, we used Maximum Likelihood (ML) and Bayesian Inference (BI) by IQ–TREE v. 2.2.0 (Nguyen et al. 2014) and MrBayes v. 3.2.7a (Ronquist et al. 2012) in the Phylosuite application (Zhang et al. 2019, Xiang et al. 2023), respectively. ModelFinder v. 2.2.0 (Kalyaanamoorthy et al. 2017) was used to select the best–fitting model of evolution, based on the Bayesian Information Criterion (BIC). Maximum-Likelihood inference was performed under the TIM+F+I+I model, which was selected as the best-fit substitution model. We applied 1,000 bootstrap pseudoreplicates with the ultrafast bootstrap approximation algorithm (UFBoot) (Agung et al. 2021), where nodes, having values 95 and above, were considered highly supported (Minh et al. 2013). Bayesian inference was conducted under the GTR+F+I model, following the approach of Agung et al. (2021). Nodes with Bayesian posterior probabilities (BPP) of 0.95 and above were considered highly supported (Huelsenbeck et al. 2001, Wilcox 2002). Uncorrected pairwise divergences were calculated by MEGA 7 (Kumar et al. 2016). The process of phylogenetic analysis was undertaken according to Zhou et al. (2025a).

**Morphological data**

Measurements were taken with a digital calipers to the nearest 0.01 mm under a dissecting microscope (Jiangnan XTB–01), following Zug (2010), Grismer et al. (2013) and Luu et al. (2023).

## Taxon treatments

### Hemiphyllodactylus lungcuensis

Luu, Nguyen, Do, Pham, Hoang, Nguyen, Le, Ziegler, Grismer & Grismer, 2023

1AD077A2-5560-5E41-80B3-28C8AFC5D54A

#### Materials

- **Type status:** Other material. **Occurrence:** occurrenceID: CDC67052-116B-5965-8A53-2B53F4F9AC39; **Taxon:** scientificNameID: *Hemiphyllodactylus lungcuensis*; **Location:** country: China; stateProvince: Yunnan Province; county: Funing county; locality: Lida Town, Funing County,Yunnan Province; verbatimElevation: 1233 m; verbatimCoordinates: 23°30'56"N, 105°32'18"E; **Event:** fieldNumber: LPSZDR0141; eventRemarks: collected by Xiuyan Li, Guangbin Liu on 10 August 2025

#### Description

**Description of the specimen from China.** Adult male, SVL 36.22 mm, flattened; longitudinal liver incision on venter. Head triangular, HL/SVL 0.29; dorsal head scales small, granular; 5 supralabials, lower two largest; 2 internasals forming isosceles triangle; mental circular; 9 chin scales between 2^nd^ and 3^rd^ infralabial sutures; gulars rounded, non-overlapping. Snout short (SnW 1.41 mm, SnW/HL 0.14); eye small (ED 2.34 mm, ED/HL 0.23). Body slender (TrunkL/SVL 0.45); dorsals granular, 17 in one eye diameter; ventrals flat, 9 in one eye diameter; limbs granular. Finger I vestigial, clawless, rectangular lamellae; Fingers II–V well developed; proximal lamellae undivided rectangular, distal divided, angular, U-shaped, terminal rounded. Digital formulae 4-5-5-5 (fore) 4-5-5(6)-5 (hind). Femoral-precloacal pores 23, continuous; white cloacal spur each side. Tail TL/SVL 0.96; dorsal caudals larger than body/head scales, smaller than subcaudals, ventrals large, flat.

#### Diagnosis

**Updated diagnosis**: Body size small; postmentals distinctly enlarged; digital lamellae 4(3)-4(5)-5(4)-4(5) (fore), 4-5(6)-5(6)-5 (hind); males with 17–25 continuous precloacal pores; single cloacal spur in both sexes; no enlarged subcaudals; dorsum light-brown to sandy, irregular dark-brown streaks; distinct dark postorbital stripe to neck base; flanks with uneven dark streaks to tail base; cream V-shaped postsacral mark with forward arms; venter pale yellow; tail light brown, contrasting with body, bearing ten alternating white and dark bands.

#### Distribution

This species is distributed in the type locality of Lung Cu Commune, Dong Van District, Ha Giang Province, Vietnam. In China, this species is only known from one location in Lida Town (Funing County).

#### Ecology

The specimen was discovered at 24:00 h on a karst boulder approximately 1.5 m above the ground on a hillside; *Hemidactylus bowringii* and two *Hemiphyllodactylus* species were also collected from the same rock. When it was illuminated, *Hemiphyllodactylus lungcuensis* crept into a crevice in the rock; when startled, it jumped off the boulder.

#### Colouration in life

**Figure 2.**
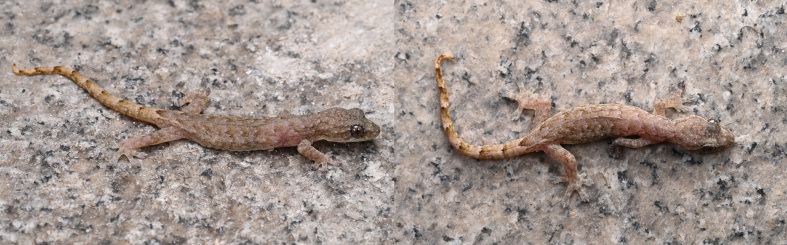
*Hemiphyllodactylus lungcuensis* (LPSZDR0141) in life.

**Figure 3.**
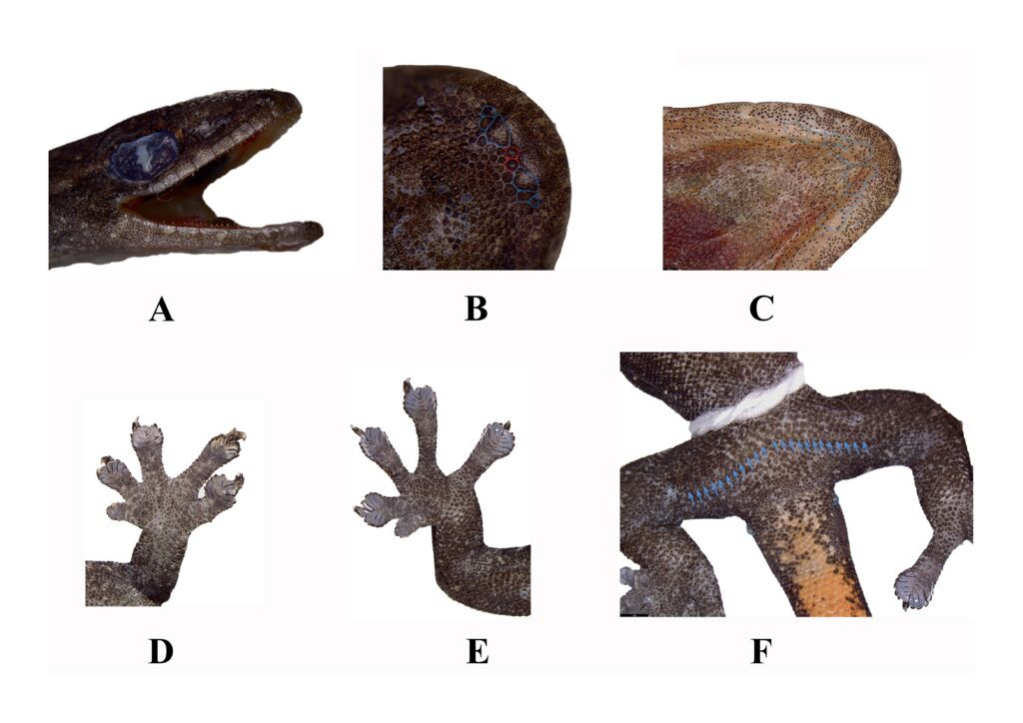
*Hemiphyllodactylus lungcuensis* (LPSZDR0141) displaying: **(A)** in lateral view of the head; **(B)** the dorsal view of the head, blue lines indicate chin scales, red lines indicate SnS; **(C)** the ventral view of the head, blue lines indicate chin scales; **(D, E)** view of lamellae formula counting on fingers and feet I to V; **(F)** the ventral view. Blue arrow indicates femoral and precloacal pores.

Dorsum of head and body grey; dark stripe from posterior corner of eye to tail base, continued on venter as lateral band; back with two dark stripes bearing irregular dark-brown and pale spots; limbs grey above, irregular dark-brown spots; tail light brown, contrasting with body, ten alternating white-dark bands; ventral tail orange-red; chin and limb venter cream-grey (Figs 2, 3).

#### Suggested Chinese common name

According to the type locality Lung Cu Commune, Dong Van District, Ha Giang Province, Vietnam, we recommend 龙古半叶趾虎 (Pinyin: lóng gǔ bàn yè zhǐ hǔ) as the Chinese common name of this species.

#### Analysis

**Figure 4.**
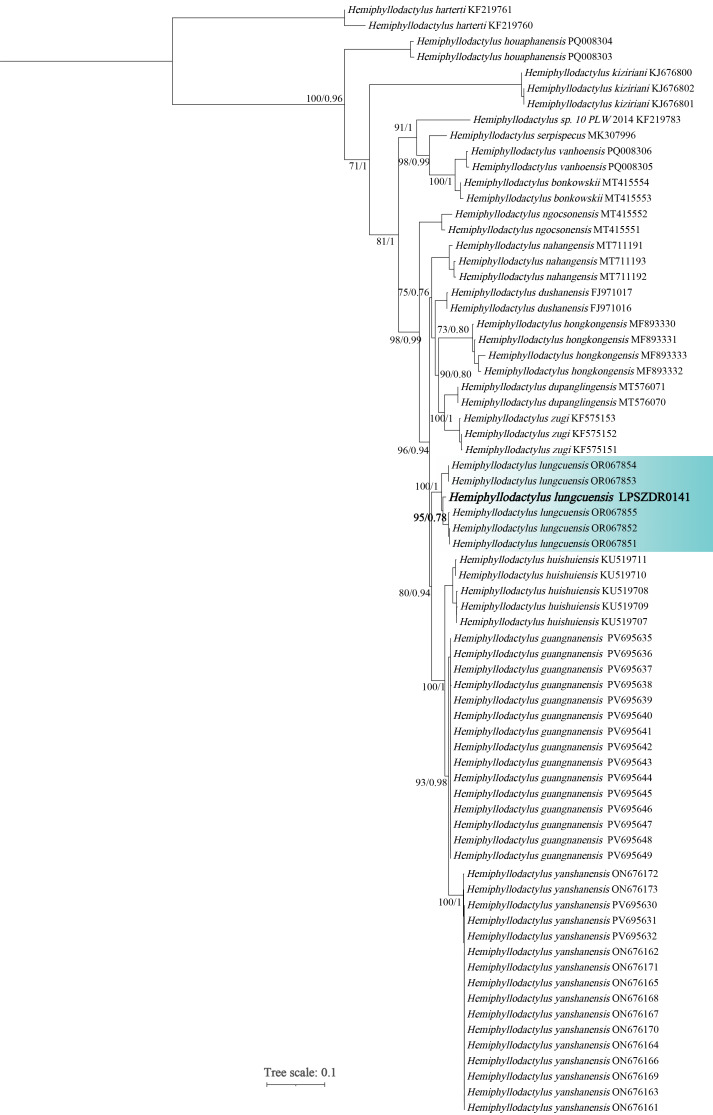
Phylogenetic tree by Maximum Likelihood estimate, based on the 1038 bp mitochondrial ND2 gene. Numbers by the nodes indicate ML bootstrap support values and posterior probability values of the BI, respectively.

The morphological measurements of the specimen from China are presented in Table 2. No obvious morphological differences were observed between the Chinese specimen and those described by Luu et al. (2023), except for minor variations, such as its having more lamelar formulae on hands and toes II–V (total lamelar formulae hands II–V = 16–17 vs. 19; total lamelar formulae on toes II–V = 19 vs. 20–21). Genetically, Bayesian Inference and Maximum Likelihood phylogeny topology were similar to Zhou et al. (2025a), with slightly different supports for some nodes, but that is not part of the scope of this present report. The sequence of the specimen from China clustered with the specimens of *H. lungcuensis* from the type locality with strong support (Fig. 4). The genetic distance (uncorrected p–distance) between the specimen from China and the other specimens of *H. lungcuensis* was 1.7% – 2.0% (Table 3).

## Discussion

Our study identifies the specimen from Yunnan Province as *Hemiphyllodactylus lungcuensis* and documents the first record of this species in China. With this addition, the Chinese *Hemiphyllodactylus* now comprises 24 species, nine of which belong to clade 7. It is noteworthy that, during our two surveys at the same location, we only collected a single specimen of *H. lungcuensis*, whereas we observed another unnamed *Hemiphyllodactylus* species and one *Hemidactylus* (*Hemidactylus bowringii*) occurring syntopically. This assemblage underscores the extraordinary diversity of *Hemiphyllodactylus* in Yunnan and mirrors patterns we have observed in Lvchun County (*Hemiphyllodactylus jinpingensis* and *H. simaoensis*) and Yuanyang County (*H. jinpingensis* and *H.* sp.). The mechanisms permitting such fine-scale sympatry remain uninvestigated; targeted fieldwork and comprehensive biogeographical analyses are needed to clarify the processes underlying this remarkable co-existence.

In China, *Hemiphyllodactylus lungcuensis* is currently known only from Funing County on the Sino-Vietnamese border, less than 30 km from the type locality in Vietnam. Vietnamese specimens were all taken from karst boulders and our single individual from Lida Town was likewise found on exposed rock, indicating a clear preference for bare lithic surfaces. In contrast, two congeners that occur in the same county and are phylogenetically close to *H. lungcuensis* were repeatedly collected inside human settlements. This pronounced niche divergence may have facilitated speciation. Nevertheless, virtually nothing is known about the ecology of any *Hemiphyllodactylus* species and we cannot yet rule out the possibility that *H. lungcuensis* could also exploit anthropogenic habitats. Detailed studies on its reproductive biology and physiological ecology are urgently needed. The recent flurry of amphibian and reptile discoveries along the Sino-Vietnamese frontier — *Ichthyophis yangi*, *Cyrtodactylus gulinqingensis*, *Rhacophorus hujianshengi*, *Paramesotriton malipoensis*, *Raorchestes malipoensis*, *Raorchestes hekouensis*, *Amolops shihaitaoi*, *Theloderma hekouense*, *Theloderma khoii* and *Zhangixalus franki*, amongst others — highlights the extensive survey gaps in this region; only sustained field effort can prevent “conservation vacuums” and the cryptic extinctions they entail.

### Ethical statement

The ethics Committee of Kunming Institute of Zoology, Chinese Academy of Sciences, approved the study and provided ethics permission (no. SMKx-20191221-216).

## Supplementary Material

XML Treatment for Hemiphyllodactylus lungcuensis

## Figures and Tables

**Table 1.** List of specimens used for phylogenetic analyses in this study.

**Species**	**Catalogue no.**	**Location**	**GenBank no.**
*H. banaensis*	ITBCZ 2450	Ba Na-Nui Chua, Vietnam	KF219783
*H. bonkowskii*	IEBR 4694	Hang Kia-Pa Co NR, Mai Chau, Hoa Binh, Vietnam	MT415553
*H. bonkowskii*	IEBR 4695	Hang Kia-Pa Co NR, Mai Chau, Hoa Binh, Vietnam	MT415554
*H. dupanglingensis*	CSUFT 00401	Dupangling, Hunan, China	MT576070
*H. dupanglingensis*	CSUFT 00405	Dupangling, Hunan, China	MT576071
*H. hongkongensis*	SYS r001728	Aberdeen Country Park, Hong Kong	MF893330
*H. hongkongensis*	SYS r001729	Aberdeen Country Park, Hong Kong	MF893331
*H. hongkongensis*	SYS r001730	Aberdeen Country Park, Hong Kong	MF893332
*H. hongkongensis*	SYS r001735	Aberdeen Country Park, Hong Kong	MF893333
*H. houaphanensis*	VNUF R.2020.16	Sa Kok, Hiem, Houaphan, Laos	PQ008303
*H. houaphanensis*	VNUF R.2020.17	Sa Kok, Hiem, Houaphan, Laos	PQ008304
*H. huishuiensis*	NJNUh00736	Huishui, Guizhou, China	KU519708
*H. huishuiensis*	NJNUh00851	Huishui, Guizhou, China	KU519707
*H. huishuiensis*	NJNUh00852	Huishui, Guizhou, China	KU519709
*H. huishuiensis*	NJNUh00857	Huishui, Guizhou, China	KU519710
*H. huishuiensis*	NJNUh00858	Huishui, Guizhou, China	KU519711
*H. kiziriani*	IEBR A.2014.3	Luang Prabang, Laos	KJ676800
*H. kiziriani*	IEBR A.2014.4	Luang Prabang, Laos	KJ676801
*H. kiziriani*	IEBR A.2014.5	Luang Prabang, Laos	KJ676802
*H. lungcuensis*	VNUF R.2021.01	Lung Cu, Dong Van, Ha Giang, Vietnam	OR067852
*H. lungcuensis*	IEBR R.5151	Lung Cu, Dong Van, Ha Giang, Vietnam	OR067853
*H. lungcuensis*	VNUF R.2021.03	Lung Cu, Dong Van, Ha Giang, Vietnam	OR067851
*H. lungcuensis*	VNUF R.2023.01	Lung Cu, Dong Van, Ha Giang, Vietnam	OR067854
*H. lungcuensis*	VNUF R.2023.02	Lung Cu, Dong Van, Ha Giang, Vietnam	OR067855
*H. nahangensis*	IEBR 4741	Trung Phin, Sinh Long, Na Hang, Tuyen Quang, Vietnam	MT711191
*H. nahangensis*	IEBR 4742	Trung Phin, Sinh Long, Na Hang, Tuyen Quang, Vietnam	MT711192
*H. nahangensis*	IEBR 4743	Trung Phin, Sinh Long, Na Hang, Tuyen Quang, Vietnam	MT711193
*H. ngocsonensis*	IEBR 4689	Ngoc Son-Ngo Luong Nr, Lac Son, Hoa Binh, Vietnam	MT415551
*H. ngocsonensis*	IEBR 4690	Ngoc Son-Ngo Luong Nr, Lac Son, Hoa Binh, Vietnam	MT415552
*H. serpispecus*	NUOL 00476	Tham Ngou Leium Cave, Viengxay, Houaphanh, Laos	MK307996
*H. vanhoensis*	VNUF R.2022.07	Tan Xuan, Van Ho, Son La, Vietnam	PQ008305
*H. vanhoensis*	VNUF R.2022.09	Tan Xuan, Van Ho, Son La, Vietnam	PQ008306
*H. yanshanensis*	KIZ062101	Yunnan, China	ON676172
*H. yanshanensis*	KIZ062102	Yunnan, China	ON676173
*H. yanshanensis*	KIZ062090	Yunnan, China	ON676161
*H. yanshanensis*	KIZ062091	Yunnan, China	ON676162
*H. yanshanensis*	KIZ062092	Yunnan, China	ON676163
*H. yanshanensis*	KIZ062093	Yunnan, China	ON676164
*H. yanshanensis*	KIZ062094	Yunnan, China	ON676165
*H. yanshanensis*	KIZ062095	Yunnan, China	ON676166
*H. yanshanensis*	KIZ062096	Yunnan, China	ON676167
*H. yanshanensis*	KIZ062097	Yunnan, China	ON676168
*H. yanshanensis*	KIZ062098	Yunnan, China	ON676169
*H. yanshanensis*	KIZ062099	Yunnan, China	ON676170
*H. yanshanensis*	KIZ062100	Yunnan, China	ON676171
*H. dushanensis*	isolate_N1	Yunnan, China	FJ971016
*H. dushanensis*	isolate_N2	Yunnan, China	FJ971017
*H. zugi*	ZFMK 94782	Ha Lang, Cao Bang, Vietnam	KF575153
*H. zugi*	IEBR A.2013.20	Ha Lang, Cao Bang, Vietnam	KF575151
*H. zugi*	IEBR A.2013.21	Ha Lang, Cao Bang, Vietnam	KF575152
*H. harterti*	LSUHC10383	Bukit Larut, Malaysia	KF219760
*H. harterti*	LSUHC10384	Bukit Larut, Malaysia	KF219761
*H. yanshanensis*	KIZ2023Z174	Yunnan, China	PV695630
*H. yanshanensis*	KIZ2023Z175	Yunnan, China	PV695631
*H. yanshanensis*	KIZ2023Z173	Yunnan, China	PV695632
*H. huishuiensis*	KIZ2023Z171	Guizhou, China	PV695633
*H. huishuiensis*	KIZ2023Z172	Guizhou, China	PV695634
*H. guangnanensis*	KIZ2023Z230	Yunnan, China	PV695635
*H. guangnanensis*	KIZ2023Z217	Yunnan, China	PV695636
*H. guangnanensis*	KIZ2023Z218	Yunnan, China	PV695637
*H. guangnanensis*	KIZ2023Z225	Yunnan, China	PV695638
*H. guangnanensis*	KIZ2023Z224	Yunnan, China	PV695639
*H. guangnanensis*	KIZ2023Z2181	Yunnan, China	PV695640
*H. guangnanensis*	KIZ2023Z220	Yunnan, China	PV695641
*H. guangnanensis*	KIZ2023Z226	Yunnan, China	PV695642
*H. guangnanensis*	KIZ2023Z222	Yunnan, China	PV695643
*H. guangnanensis*	KIZ2023Z214	Yunnan, China	PV695644
*H. guangnanensis*	KIZ2023Z223	Yunnan, China	PV695645
*H. guangnanensis*	KIZ2023Z221	Yunnan, China	PV695646
*H. guangnanensis*	KIZ2023Z219	Yunnan, China	PV695647
*H. guangnanensis*	KIZ2023Z227	Yunnan, China	PV695648
*H. guangnanensis*	KIZ2023Z229	Yunnan, China	PV695649
* H. * *** lungcuensis ***	**LPSZDR0141**	**Funing County, Yunnan, China**	**PX398552**

**Table 2.** Morphological characters of *Hemiphyllodactylus lungcuensis* by Luu et al. (2023) and specimen from Funing County, China.

**Characteristics**	**Luu et al. (2023)**	**LPSZDR0141**
SVL	35.3–44.2	36.22
TL	34.7–42.7	34.95
TrunkL	17.0–23.1	16.38
HL	8.5–11.6	10.36
HW	6.9–8.0	6.16
ED	1.8–2.5	2.34
SnEye	3.6–5.0	4.08
NarEye	2.9–3.6	3.32
SnW	1.4–2.1	1.41
CN	2–3	3
IS	1–3	2
SL	11–12	11
IL	9–11	9
Chin	8–10	9
DS	12–17	17
VS	6–11	9
FL1	3–4	4/4
TL1	4	4/4
FL2–5	4–(3)4–5–(4)4	4–5–5–5
TL2–5	4–5–5–5	4–5(6)–6–5
Total number of precloacal and femoral pores	17–25	23
Number of cloacal spurs	1–2	1
Precloacal and femoral pore series separate (1) or continuous (0)	0	0
Subcaudals enlarged, plate-­like	0	0
Dark dorsal transverse blotches	Yes	Yes
Dark postorbital stripe	Yes	Yes
Dorsolateral light-coloured spots on trunk	Yes	Yes
Postsacral marking anteriorly projecting arms	Yes	Yes

**Table 3.** **Table 3.** The uncorrected *p*–distance amongst the *Hemiphyllodactylus lungcuensis* from different locations, based on mitochondrial ND2 gene fragments.

**Species name (no.)**	**1**	**2**	**3**	**4**	**5**	**6**
**1. *H. lungcuensis* (LPSZDR0141)**						
2. *H. lungcuensis* (OR067851)	1.8					
3. *H. lungcuensis* (OR067852)	1.8	0				
4. *H. lungcuensis* (OR067853)	2.0	2.6	2.6			
5. *H. lungcuensis* (OR067854)	2.0	2.6	2.6	0		
6. *H. lungcuensis* (OR067855)	1.7	0.3	0.3	2.3	2.3	
